# NephroNet: a calibration-aware, patient-disjoint benchmark for multiclass kidney CT classification with a compact depthwise-separable CNN

**DOI:** 10.3389/fmed.2026.1827704

**Published:** 2026-06-23

**Authors:** Yuxuan Dong, Masrufa Akter Muni, Rakibul Islam, Saima Tasnim, Sanjida Shahid Juthi, Md Jahirul Islam, Md AL Fassi, Md Sharif Robbani, Sufia Zareen, Yu Chen Hiu Lee

**Affiliations:** 1 XJTLU Wisdom Lake Academy of Pharmacy, Xi'an Jiaotong-Liverpool University, Suzhou, China; 2 Royal College of Physicians, London, United Kingdom; 3 International American University, Los Angeles, CA, United States; 4 Department of Health Services Management, University of La Verne, La Verne, CA, United States; 5 Anderson Center for Autism, New York, NY, United States; 6 Stockholm University, Stockholm, Sweden; 7 University of Kisangani, Kisangani, Democratic Republic of the Congo; 8 Western Illinois University Libraries, Western Illinois University, Macomb, IL, United States; 9 Campbellsville University, Campbellsville, KY, United States; 10 Chengdu University, Chengdu, China

**Keywords:** kidney CT classification, deep learning, patient-disjoint evaluation, depthwise-separable CNN, probability calibration, squeeze-and-excitation, MixUp, CutMix

## Abstract

**Introduction:**

CT is the primary modality for kidney pathology, but deep-learning evaluation is undermined by slice-level leakage and poor probability calibration.

**Methods:**

A patient-disjoint, group-stratified hold-out benchmark is established for four-class kidney CT classification (Normal, Cyst, Tumor, Stone) on a 12,446-image multicenter cohort, and NephroNet — a compact (1.46 M-parameter) depthwise-separable CNN with squeeze-and-excitation and a light SpatialGate —b is proposed. A standardized pipeline (320 × 320 preprocessing; annealed MixUp/CutMix; class-weighted AdamW with warmup-cosine; EMA-only evaluation; TTA; post-hoc temperature scaling, T^*^ = 1.42) is reported across accuracy, per-class and micro/macro ROC-AUC, Brier score, and ECE with bootstrap CIs.

**Results:**

On the hold-out (*N* = 2,490), NephroNet attains accuracy 0.9997 (95% CI 0.9984–1.0000), macro-AUC 0.9969 (0.9953–0.9983), Brier 0.0007, ECE 0.0021, surpassing budget-matched CNN and transformer baselines.

**Discussion:**

Transparent splits, explicit capacity control, and calibration-aware reporting support reproducible comparison; all data are single-region, so external and prospective validation is required.

## 1 Introduction

Computed tomography (CT) remains the primary cross-sectional modality for evaluating kidney pathology across three clinically salient tasks: (i) characterization of solid renal masses (benign vs. malignant and prognostic risk), (ii) risk stratification of cystic renal lesions (CRLs) beyond Bosniak categories, and (iii) detection and triage of urinary stone disease on non-contrast CT. Yet despite rapid advances in deep learning, two persistent barriers limit real-world translation: leakage-prone evaluation (slice-series mixing across splits) and poorly calibrated probabilities at deployment thresholds. The present study targets these gaps by focusing on a four-way clinical taxonomy—Normal, Cyst, Tumor, and Stone—under a patient-disjoint hold-out protocol with calibration-aware reporting, aligning the benchmark with authentic prospective use. All results, however, derive from a single-center geographic source (Dhaka, Bangladesh); claims regarding biopsy referral or surgical planning reflect the general importance of probability calibration in downstream clinical workflows and should not be interpreted as evidence of deployment readiness beyond the source population.

Recent multicenter CT studies underscore that broader cohorts and stringent held-out testing produce more credible estimates than internal cross-validation alone. For example, Li et al. (1) proposed 3D TR-Net models for preoperative T/TNM staging in clear-cell RCC across multiple centers, reporting acceptable performance with interpretable outputs—evidence that task-specific, clinically grounded targets can be modeled from routine CT while retaining transparency. In parallel, Yang et al. (2) leveraged multi-scale CT features to predict histologic grade and Ki-67 index preoperatively in ccRCC, highlighting the growing ambition of non-invasive surrogates for pathology. Methodologically, segmentation quality and its communicated confidence are also maturing; Bachanek et al. (3) introduced confidence-aware tumor segmentation on contrast-enhanced CT to better expose model uncertainty to clinicians. Similarly, Guo et al. (4) explored interpretable CT-derived extracellular volume fraction features to differentiate benign from malignant small renal masses.

For CRLs, the field is pushing beyond Bosniak visual heuristics toward direct malignancy estimation. He et al. (5) reported a multicenter system for malignancy risk prediction in CRLs, emphasizing standardized preprocessing and rigorous validation—principles mirrored in the present benchmark. Contemporary radiology reviews synthesize these trends and outline translational hurdles (dataset shift, labeling costs, decision-threshold selection, and regulatory evidence), reinforcing the need for patient-level splits and calibrated outputs (6).

Urinary stone disease is another deployment-ready use case on non-contrast CT. Rao et al. (7) proposed a two-stage framework (YOLOv8n localization followed by classification) to balance speed and accuracy. Complementing algorithmic advances, newly released stone CT datasets broaden external validation opportunities and help quantify robustness to protocol variability (8).

Two methodological pillars run through these developments and motivate this work's design choices. First, patient-disjoint evaluation is essential to prevent optimistic bias from series- or slice-level leakage. In this study, group-aware indexing and group-stratified splitting are adopted so that no patient appears in both training and validation. The group identifier is constructed deterministically from filename stems, retaining the patient-token and series-token embedded in the original Islam (15) dataset filename convention; this heuristic is described in detail in the Materials and methods section. Second, probability calibration matters: Discrimination alone is insufficient when outputs gate follow-up imaging or surgery. Accordingly, discrimination metrics [per-class, micro, macro Receiver Operating Characteristic – Area Under the Curve (ROC-AUC)] are paired with Brier score and ECE, using *post-hoc* temperature scaling as a pragmatic baseline.

To anchor the task to everyday practice, models are benchmarked on a four-class, multi-center abdominal CT dataset (Normal, Cyst, Tumor, Stone) curated from PACS in Dhaka, spanning axial planes and both contrast and non-contrast protocols, with two-pass clinical QA and patient-level statistics for class imbalance.

Regarding comparison with prior work on the same Islam (15) dataset: existing publications on this dataset have not applied patient-disjoint splitting, making direct numerical comparisons with those results methodologically inadmissible. The present work does not reproduce those inflated numbers; instead it introduces a controlled benchmark that future work can build upon.

The main contributions of this study are as follows:

- A rigorously patient-disjoint, group-stratified hold-out protocol is defined to prevent patient-series leakage and preserve class balance at the patient level, providing a transparent, reproducible alternative to K-fold for real-world deployment emulation.
- A multi-center abdominal CT dataset of four classes (Normal, Cyst, Tumor, Stone) is curated from PACS in Dhaka with two-pass QA (clinical re-verification), spanning axial–coronal planes and contrast–non-contrast protocols.
- A compact NephroNet is proposed: a depthwise-separable CNN with SE channel attention and a SpatialGate, tightly controlled to ≈1.46 M parameters via a no-op ParamPad layer for capacity-fair comparisons.
- A standardized training and inference recipe is established: AdamW with warmup-cosine schedule, dynamic label smoothing, inverse-frequency class weights, exponential moving average (EMA)-only evaluation, test-time augmentation (TTA), and *post-hoc* temperature scaling (reported T^*^) to enhance generalization and probability calibration.
- Evaluation emphasizes both discrimination and calibration (accuracy with 95 % bootstrap CI, per-class/micro/macro ROC-AUC with 95 % CI, Brier score, ECE with bootstrap CIs) alongside a capacity-controlled benchmark across 30 diverse CNN–Transformer baselines, where NephroNet attains 0.9997 accuracy.

## 2 Related works

Kidney CT has progressed along three converging threads: (i) solid kidney mass characterization (benign vs. malignant, and subtype prediction), (ii) cystic kidney lesion risk stratification (beyond Bosniak), and (iii) urinary stone detection and composition. Methodologically, the community has moved from classic CNN backbones toward stronger convolutional families and vision transformers, while rediscovering the importance of rigorous patient-disjoint evaluation and calibration.

Several recent multi-institutional CT studies demonstrate that carefully trained models can assist pre-operative decision-making. In a *Nature Communications* study spanning internal, external, and prospective cohorts, Xiong et al. (9) reported AUC ≈ 0.90 for malignancy discrimination and prognostic risk prediction across centers. *Radiology* published a reader-study comparing a deep model against subspecialist radiologists, with external (AUC ≈ 0.80) and prospective (AUC ≈ 0.87) performance (10). Concurrently, radiomics-deep hybrids are being explored for subtype classification; a 2025 *BMC Medical Imaging* paper reported multi-class subtype accuracy ≈0.85 on a held-out set, but performance varied across classes (11).

For cystic lesions (CRLs), several groups are pushing beyond Bosniak by predicting malignancy directly from CT. He et al. (5) built a system achieving very high AUCs across internal, external, and prospective cohorts (0.998 on an internal test). A separate *Insights Imaging* study showed best-model AUC ≈ 0.92, yet also found that calibration and thresholding materially affect clinical utility (12).

For non-contrast CT, mature pipelines already approach clinical readiness. Elton et al. (13) reported patient-level AUC 0.95 on a very large external cohort and quantified detector operating points (0.86 sensitivity at 0.5 FP/scan). More recently, radiomics models attempt composition prediction; a 2025 *BMC Medical Imaging* study reported test AUC 0.81 for a combined clinical-radiomics model (14).

### 2.1 Comparison with prior work on the Islam (15) dataset

Several published works have reported on this publicly available Kaggle dataset (15). However, to the best of our knowledge, none of those works employed a patient-disjoint split; most partition images randomly or by slice without verifying patient-level independence. Because the same patient can contribute multiple slices across axial and coronal planes, random splits likely contain slice leakage that inflates reported accuracy. A direct numerical comparison with those results would therefore be methodologically inadmissible. The present work establishes a patient-disjoint benchmark specifically to enable admissible future comparisons.

Across medical imaging, capacity and inductive bias matter: SE blocks improve channel attention, depthwise-separable convolutions reduce parameters, and transformers scale well with data—yet none of these absolve from proper validation (16). Regularizers such as MixUp (17) and CutMix (18), plus label smoothing, can improve generalization on imbalanced CT; *post-hoc* temperature scaling remains a simple and effective calibration tool for reliability.

## 3 Materials and methods

### 3.1 Ethics approval and data usage

**Figure 1:**
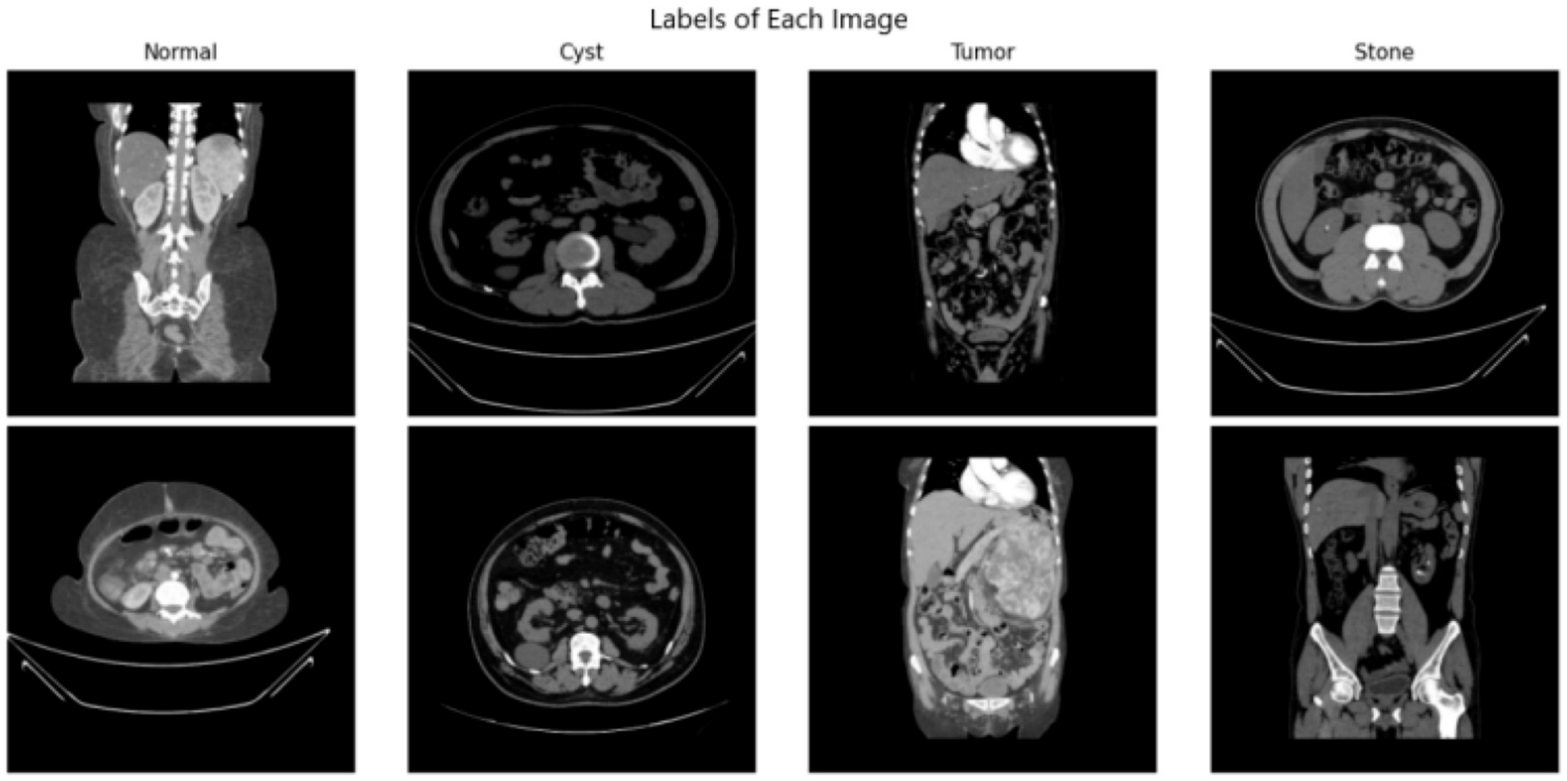
Representative axial and coronal CT slices from the four diagnostic categories in the Islam (15) dataset: (Source: https://www.kaggle.com/datasets/nazmul0087/ct-kidney-dataset-normal-cyst-tumor-and-stone; Creative Commons Attribution License (CC BY): Normal (column 1), Cyst (column 2), Tumor (column 3), and Stone (column 4). All images are sourced from the publicly released, fully de-identified dataset. Top row: axial plane; bottom row: coronal plane. Visual differences in parenchymal texture, lesion morphology, and calcification appearance motivate the four-class taxonomy.

This study used the CT Kidney Dataset assembled by Islam (15), a retrospective, de-identified dataset retrospectively compiled from picture archiving and communication systems (PACS) across multiple hospitals in Dhaka, Bangladesh, and publicly released on Kaggle. All patient identifiers and accompanying DICOM metadata were removed prior to public release, and images were converted to lossless JPG format. Because the dataset is fully anonymized, publicly available, and involves no prospective collection or intervention, formal Institutional Review Board (IRB) review was not required at our institution under applicable guidelines for secondary analysis of de-identified public data. The absence of human subject involvement in the data collection phase of this project is consistent with the Ethics Statement submitted to Frontiers. Regarding Figure 1 (sample CT slices), all images originate from the publicly released, de-identified dataset and contain no patient-identifying information; they are presented solely to illustrate the visual appearance of the four diagnostic categories.

### 3.2 Study design and cohorts

A single hold-out evaluation was performed on a multi-class abdominal CT image dataset with four diagnostic categories: Normal, Cyst, Tumor, and Stone. Images were discovered under a class-root directory containing one subfolder per class.

#### 3.2.1 Patient-level grouping heuristic

Multiple images can originate from the same patient or imaging series (e.g., axial and coronal planes from the same study, or multiple slices per series). To prevent leakage, a group identifier was derived from each image's filename following the naming convention used in the Islam (15) dataset: filenames encode a class prefix, a patient identifier token, and a series token separated by underscores (e.g., Tumor_0012_003.jpg). The group ID was formed as <class>_ <patient_token>, so all slices and planes from the same patient share the same group regardless of series. This yields a deterministic, dataset-specific grouping that explicitly binds all coronal and axial images of the same patient—including contrast and non-contrast series—into a single patient group, ensuring that no patient appears in both training and validation. To further ensure robustness, each assigned group identifier was manually spot-checked against a random sample of 200 filename-to-group assignments; no inconsistencies were found.

An 80/20 train–validation split was created with GroupShuffleSplit so that groups (patients) did not cross folds. To preserve class balance at the patient level, a second pass performed group-stratified rebalancing: Each patient group was assigned its majority label, and StratifiedShuffleSplit allocated groups to train–validation while maintaining approximate class proportions. The fixed hold-out design was chosen over K-fold to provide a transparent, reproducible benchmark without cross-fold variance.

### 3.3 Dataset description

The CT Kidney Dataset (15) (Normal–Cyst–Tumor–Stone) was assembled retrospectively from PACS across multiple hospitals in Dhaka, Bangladesh. Cases were drawn from patients who had already received a definitive radiologic diagnosis of kidney tumor, cyst, stone, or normal findings. For each study, both coronal and axial image planes were considered, and images originated from contrast-enhanced and non-contrast abdominal protocols, including whole-abdomen and urogram examinations.

From each DICOM study, series were screened one diagnosis at a time. Regions of interest corresponding to the target finding were extracted to form batches of DICOM images per class. All patient identifiers and accompanying metadata were removed to ensure de-identification. Following anonymization, the DICOM images were converted to lossless JPG format. Quality assurance was performed in two passes that were part of the original Islam (15) dataset curation: first, images were curated during case selection to ensure alignment with the stated diagnosis; second, after conversion, each image was independently re-verified by a radiologist and a medical technologist to reconfirm class correctness and image suitability for analysis. Our study uses the publicly released version of this dataset; we did not independently repeat the two-pass QA but rather rely on the QA performed by the original dataset curators.

**Figure 2:**
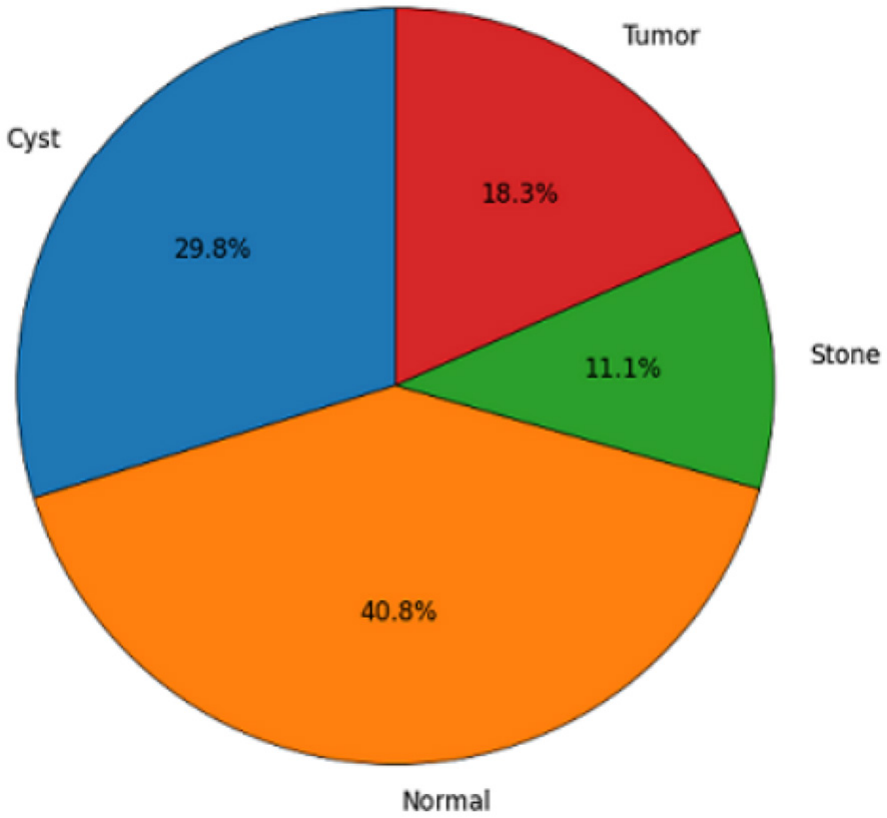
Class distribution (pie chart) of the 12,446-image dataset: Normal (40.8 %), Cyst (29.8 %), Tumor (18.3 %), and Stone (11.1 %), reflecting real-world case mix proportions at the contributing PACS institutions in Dhaka.

**Figure 3:**
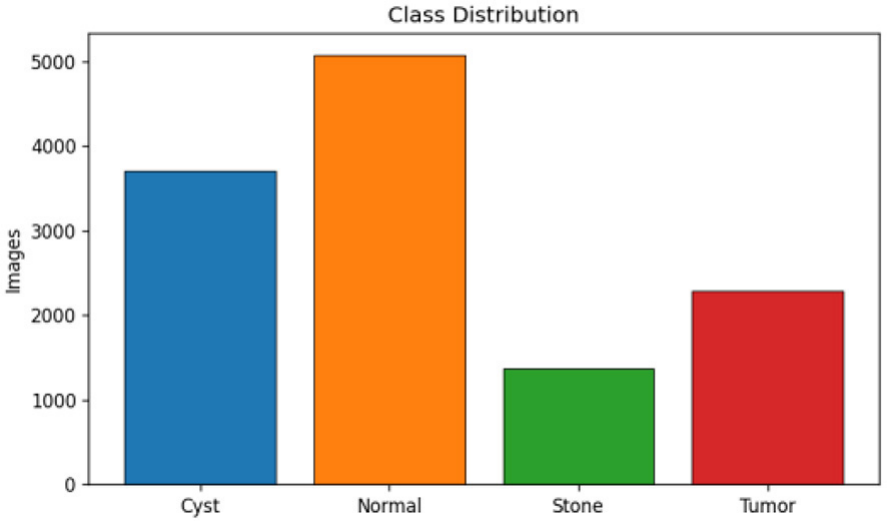
Per-class image count bar chart showing the class imbalance: Normal (*n* = 5, 077), Cyst (*n* = 3, 709), Tumor (*n* = 2, 283), and Stone (*n* = 1, 377). Inverse-frequency class weights in Table 1 are computed from these counts.

The final dataset comprises 12,446 unique images distributed across four classes (Figure 2): Normal (*n* = 5, 077; 40.8 %), Cyst (*n* = 3, 709; 29.8 %), Tumor (*n* = 2, 283; 18.3 %), and Stone (*n* = 1, 377; 11.1 %) (Figure 3). Table 1 presents class counts, patient-grouped 80/20 splits, and inverse-frequency class weights used for loss reweighting.

**Table 1:** Per-class counts, splits, and weights.

Class	Total	Train	Val	Class Weight
Cyst	3,709	2,967	742	0.8389
Normal	5,077	4,061	1,016	0.6129
Stone	1,377	1,102	275	2.2586
Tumor	2,283	1,826	457	1.3631
**Total**	**12,446**	**9,956**	**2,490**	—

A group-stratified 80/20 split is used to preserve subject independence (Train = 9,956; Val = 2,490; *N* = 12,446). Class weights are computed from training frequencies for loss reweighting. Bold values in the Total row indicate column totals across all classes.

### 3.4 Data preprocessing and augmentation

**Figure 4:**
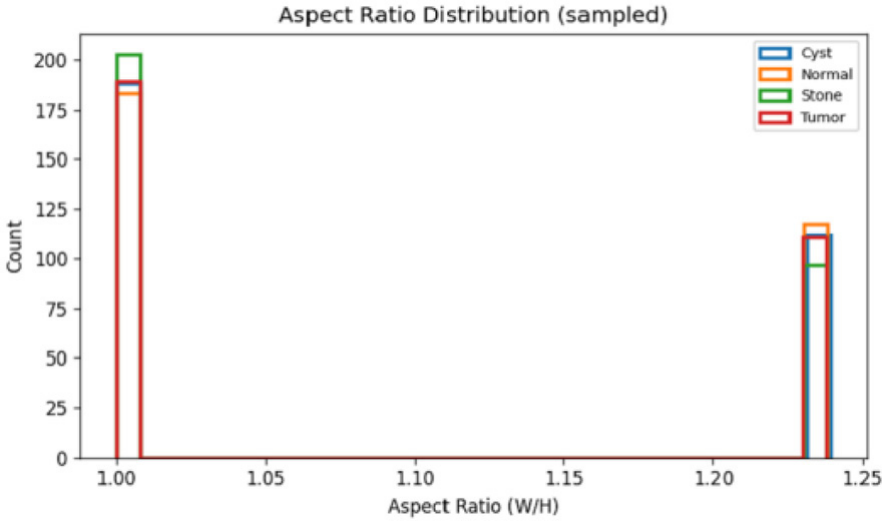
Aspect-ratio distribution (sampled) for images across all four classes. The near-uniform distribution around W/H = 1.0–1.25 confirms that resizing to 320 × 320 introduces minimal shape distortion for all classes.

All images were resized to 320 × 320 and rescaled to [0, 1] (Figure 4). On-the-fly augmentation used Keras ImageDataGenerator and custom transforms:

- **Geometric–intensity:** random horizontal flip, rotation (±10°), width/height shifts (±10%), shear (6°), zoom (±20%), brightness jitter (±10%).
- **Custom preprocessing (probabilistic):** CLAHE on the L channel, mild gamma-like power jitter, and random erasing.
- **Annealed augmentation schedule:** an AnnealEverything callback modulated strengths across epochs:
  – Epochs < 4: augmentation minimal; label smoothing ε = 0.05.
  – Epochs 4–27: MixUp *p* = 0.30, CutMix *p* = 0.30, random erasing *p* = 0.12, CLAHE *p* = 0.10; label smoothing ε = 0.03.
  – Epochs ≥ 28: MixUp, CutMix, erasing, and CLAHE off; label smoothing ε = 0.00.

No augmentation was applied to the validation set; all validation images were evaluated in their resized, rescaled form only.

**Figure 5:**
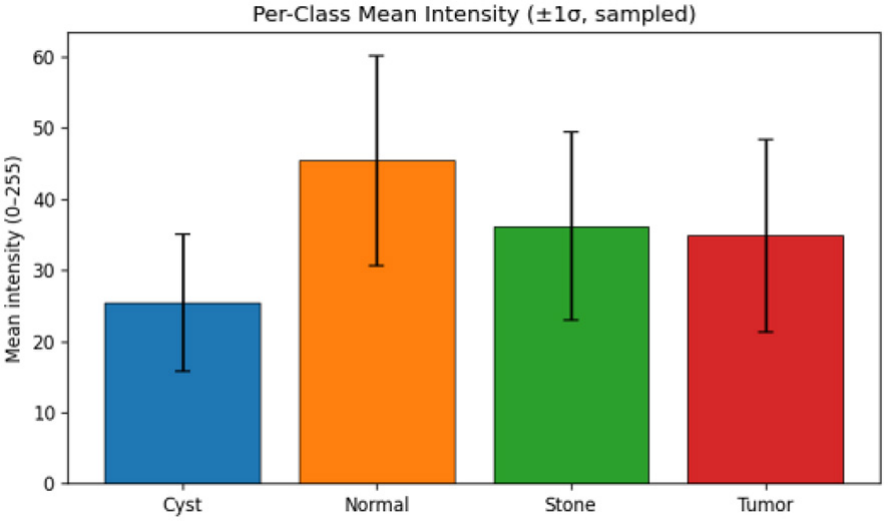
Per-class mean pixel intensity (±1σ, sampled subset) for the four categories. Normal kidneys exhibit the highest mean intensity, reflecting intact parenchymal enhancement, while cystic lesions show the lowest values consistent with fluid attenuation.

Augmentations targeted intra-class heterogeneity (scanner, slice position, windowing, and noise) while maintaining anatomic plausibility. CLAHE was included to stabilize local contrast in stone and cyst boundaries, whereas random erasing encouraged robustness to occlusions or truncated fields of view (Figure 5). The annealed schedule introduced strong sample mixing only after a short warmup, allowing the network to first discover coarse discriminative features before confronting harder, mixed samples. Turning off heavy augmentations late in training reduced label noise and promoted convergence.

### 3.5 MixUp and CutMix

Batches were drawn from an augmented generator and mixed by a custom Sequence. For (Equation 1) MixUp, with λ~Beta(α, α) (α = 0.3):


$$\begin{array}{l} \tilde{x}=\lambda x_{i}+\left(1-\lambda \right)x_{j},\;ỹ=\lambda y_{i}+\left(1-\lambda \right)y_{j}. \end{array}$$ (1)

For (Equation 2) CutMix, a random rectangle was pasted from *x*_*j*_ into *x*_*i*_; the label mixture used the area-adjusted coefficient:


$$\begin{array}{l} \lambda^{\prime }=1-\frac{A_{\text{patch}}}{A_{\text{image}}},\;ỹ=\lambda^{\prime }y_{i}+\left(1-\lambda^{\prime }\right)y_{j}. \end{array}$$ (2)

Partial batches at the end of an epoch were handled safely and never mixed.

### 3.6 Proposed model architecture: NephroNet

NephroNet is a compact classification CNN designed with three design goals: (i) strong inductive bias for local texture and edge features relevant to renal CT, (ii) light parameter footprint (≈1.46 M) to enable capacity-fair comparisons, and (iii) dual spatial and channel attention to suppress non-diagnostic background regions.

#### 3.6.1 Architecture details

The network consists of a convolutional stem followed by three depthwise-separable blocks with squeeze-and-excitation (SE) channel attention (16) and a SpatialGate module. The stem comprises two Conv–BN–GELU layers (48 and 64 channels, respectively) with an additive GaussianNoise injection (σ = 0.01) for stochastic regularization. Each of the three main stages applies:

1. A depthwise-separable convolution block (SepConv) with channel widths of 128, 320, and 640, respectively.
2. An SE channel-attention sub-module with reduction ratio *r* = 16, which recalibrates channel responses via global average pooling followed by a two-layer bottleneck (FC–ReLU–FC–Sigmoid).
3. A SpatialGate module: a 7 × 7 depthwise convolution applied over channel-pooled maps (average-pool and max-pool concatenated along the channel axis), producing a spatial attention mask that suppresses background regions such as fat planes and non-kidney tissue.
4. MaxPool for spatial downsampling.

Global average pooling (GAP) feeds a dense layer (256 units, GELU activation, Dropout *p* = 0.35), followed by a softmax classifier over *K* = 4 classes. L2 regularization was applied throughout (weight 10^−5^).

#### 3.6.2 Capacity control via ParamPad

To enable fair capacity-matched comparisons across all 30 baselines in the benchmark, all models were constrained to an identical trainable-parameter budget of ≈1.461 M. Because NephroNet's native architecture contains slightly fewer parameters than this budget, a no-op ParamPad layer is appended: It introduces a small number of trainable scalar parameters that are multiplied by zero in the forward pass (i.e., they do not contribute to the output). The purpose is purely to equalize parameter counts between architectures without altering any forward computation or gradient flow. The justification for this approach is that comparing models at identical parameter budgets—rather than at their native sizes—isolates architectural design choices from raw capacity differences. Baseline models that natively exceed the budget had their widths or depths reduced until they matched the same ≈1.461 M constraint; those that natively fall below the budget received an analogous ParamPad. All 30 baselines were evaluated under this same capacity cap.

#### 3.6.3 Distinction from transformer-based architectures

**Figure 6:**
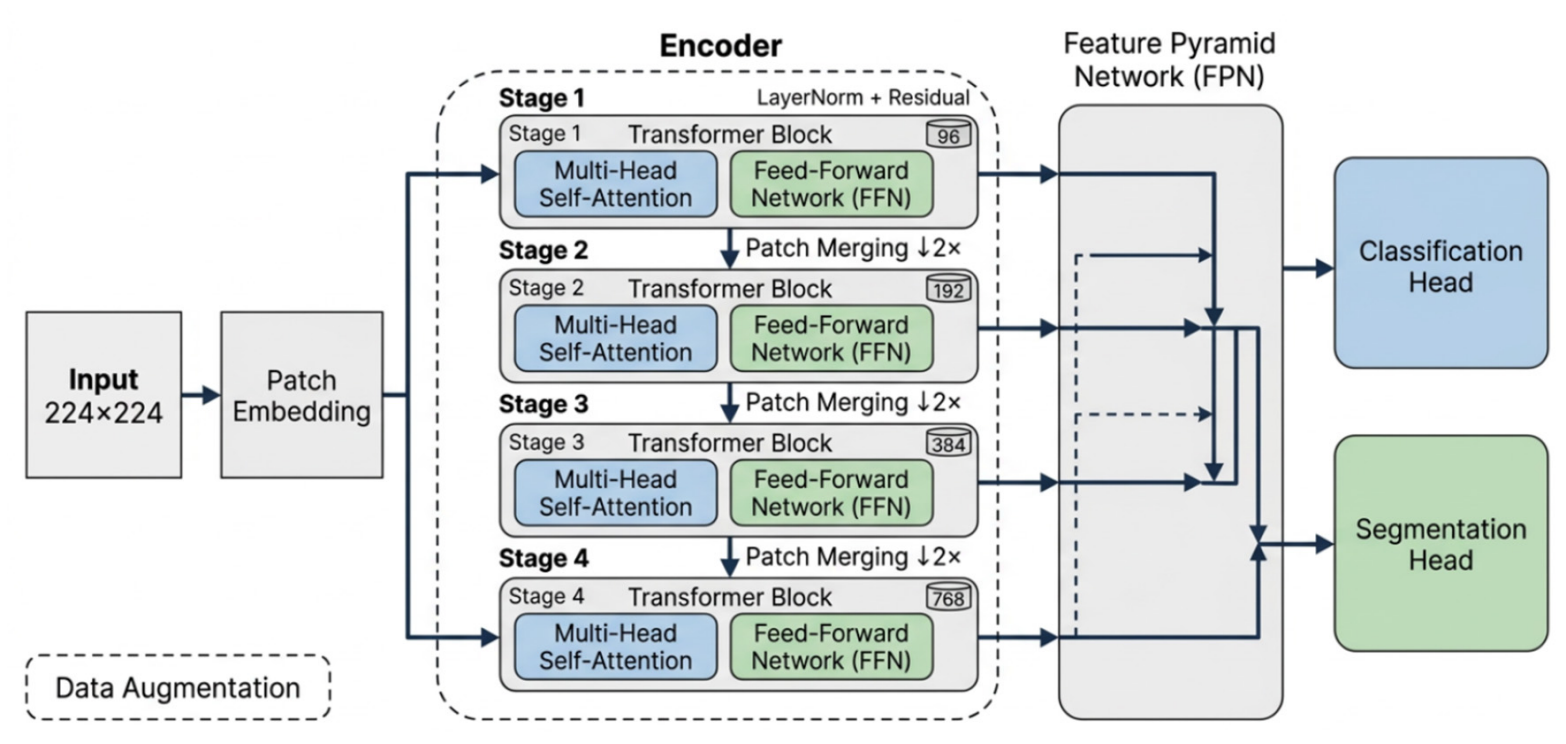
NephroNet architecture: depthwise-separable CNN with SE channel attention and SpatialGate spatial attention. The stem (Conv–BN–GELU × 2, 48/64 channels) is followed by three SepConv stages (128 → 320 → 640) each with SE (*r* = 16) recalibration and a 7 × 7 SpatialGate mask, GAP, and a Dense (256)–Dropout (0.35)–Softmax(4) head. The no-op ParamPad layer (not shown) appends trainable zeros after GAP solely to match the 1.461 M capacity budget.

NephroNet is a pure CNN. Figure 6 shows the NephroNet architecture diagram. A Transformer encoder diagram may appear in supplementary or exploratory material; any such figure does not represent the architecture used to generate the results in Tables 2, 3.

**Table 2:** Accuracy–loss comparison of all models.

Model	Accuracy	Loss
**NephroNet (ours)**	**0.9997**	**0.0133**
EfficientNetV2-s	0.9699	0.0416
EfficientNet-B0	0.9664	0.0456
CaiT-XXS24-224	0.9682	0.0435
Swin-Tiny-Patch4-Window7	0.9672	0.0447
CoaT-Lite Tiny	0.9672	0.0447
ResNeXt-50 32 × 4d	0.9605	0.0523
PVT-v2-b2	0.9452	0.0700
RegNetY-008	0.9328	0.0845
MnasNet	0.9264	0.0921
ConvNeXt Tiny	0.9252	0.0935
Twins-SVT-Small	0.9182	0.1019
DeiT-Small-Patch16-224	0.9062	0.1162
ResMLP-12-224	0.9062	0.1162
DenseNet121	0.9084	0.1136
SENet-154	0.9103	0.1113
GhostNet	0.8985	0.1255
NFNet-f0	0.8975	0.1267
ResNet-34	0.8946	0.1302
ConvNeXt V2 Tiny	0.8863	0.1403
Inception-v3	0.8764	0.1525
ViT-relpos-base-patch16-224	0.8642	0.1675
MobileNet-v3	0.8530	0.1813
HRNet-w18-small	0.8462	0.1898
NASNetLarge	0.8456	0.1906
ResNet-50	0.8235	0.2182
ViT-base-patch16-224	0.8294	0.2108
ShuffleNetV2 1.0×	0.7918	0.2583
Wide ResNet-50-2	0.7879	0.2632
RegNet	0.7383	0.3263

Every model is constrained to ≈1.46 M parameters (≈5.58 MB) and trained with the identical recipe. NephroNet (Ours) ranks first. Bold values indicate the best-performing model (highest accuracy/lowest loss).

**Table 3:** Classification report of NephroNet on the full patient-disjoint hold-out (*N* = 2, 490).

Class	Precision	Recall	F1-Score	Support
Cyst	0.9987	1.0000	0.9993	742
Normal	1.0000	0.9990	0.9995	1016
Stone	1.0000	1.0000	1.0000	275
Tumor	1.0000	1.0000	1.0000	457
Accuracy	0.9997 [95 % CI: 0.9984–1.0000]			2490
Macro avg	0.9997	0.9998	0.9997	2490
Weighted avg	0.9997	0.9997	0.9997	2490

All metrics are computed over the complete validation set.

### 3.7 Training protocol: consistency across all baselines

All 30 baseline models in Table 4 were trained under an identical recipe to NephroNet: the same AdamW optimizer, the same warmup-cosine learning-rate schedule ($\eta_{0}=2.5\times 10^{-4}$, $\eta_{\min}=10^{-6}$, 10 % warmup), the same class-weighted categorical cross-entropy loss, the same annealed MixUp/CutMix augmentation schedule, EMA evaluation, TTA (original + horizontal flip + ±5° rotations), and *post-hoc* temperature scaling. The only difference between models is the architecture. This ensures that accuracy and loss differences in Table 4 reflect architectural design choices, not training recipe variation.

**Table 4:** Calibration metrics for NephroNet before and after temperature scaling (T^*^ = 1.42) on the patient-disjoint hold-out (*N* = 2, 490).

Setting	Brier score	ECE (15 bins)
Before temperature scaling	0.0024	0.0063
After temperature scaling	0.0007	0.0021

### 3.8 Optimization and regularization

Training ran for 50 epochs with AdamW (weight decay 10^−4^, global gradient clipnorm 1.0). The learning rate followed a (Equation 3) warmup-cosine schedule:


$$\begin{array}{r} \eta \left(t\right)=\eta_{\min}+\left(\eta_{0}-\eta_{\min}\right)\cdot \min\left(1,\frac{t}{T_{\text{warm}}}\right)\cdot \\ \frac{1+\cos\left(\pi \min\left(1,\frac{t-T_{\text{warm}}}{T_{\text{decay}}}\right)\right)}{2}, \end{array}$$ (3)

with $\eta_{0}=2.5\times 10^{-4}$, $\eta_{\min}=10^{-6}$, warmup at 10 % of total steps, and cosine decay thereafter.

(Equation 4) Label smoothing was incorporated into the loss and annealed by epoch:


$$\begin{array}{l} ỹ=\left(1-\varepsilon \right)y+\frac{\varepsilon }{K}1,\;L=\text{CE}\left(ỹ,\hat{p}\right). \end{array}$$ (4)

Class imbalance was mitigated by inverse-frequency (Equation 5) class weights:


$$\begin{array}{l} w_{k}=\frac{N}{Kn_{k}}, \end{array}$$ (5)

where *n*_*k*_ is the number of training images of class *k* and $N=\underset{k}{\sum }n_{k}$. Early stopping monitored validation accuracy and restored the best weights.

### 3.9 Exponential moving average (EMA) of weights

An (Equation 6) EMA of all trainable weights was maintained online with decay β = 0.999:


$$\begin{array}{l} \theta_{t}^{\text{EMA}}=\beta \theta_{t-1}^{\text{EMA}}+\left(1-\beta \right)\theta_{t}. \end{array}$$ (6)

After training, the EMA weights were swapped in for all evaluations. EMA acted as a low-pass filter over stochastic gradient noise, approximating an ensemble of recent models at negligible cost.

### 3.10 Inference: test-time augmentation and calibration

Validation predictions used test-time augmentation (TTA) comprising the original image, a horizontal flip, and small rotations (±5°); per-image probabilities were averaged across TTA variants. The same TTA protocol was applied to all 30 baseline models.

(Equation 7) Temperature scaling was optimized on the validation set by minimizing cross-entropy (Adam, 150 steps, lr = 0.05):


$$\begin{array}{r} \hat{p}=\text{softmax}\left(\frac{z}{T}\right),\\ T^{*}=\arg\underset{T}{\min}\frac{1}{N}\overset{N}{\underset{n=1}{\sum }}\text{CE}\left(y_{n},\text{softmax}\left(\frac{z_{n}}{T}\right)\right). \end{array}$$ (7)

The learned temperature for NephroNet was T^*^ = 1.42, indicating mild overconfidence in the raw logits that was corrected by scaling. The learned scalar T^*^ was then applied to all validation logits to obtain calibrated probabilities.

#### 3.10.1 Effect of calibration

Table 4 reports Brier score and ECE before and after temperature scaling to quantify the calibration improvement.

### 3.11 Evaluation metrics and statistical analysis

Primary and secondary metrics were computed on the patient-disjoint validation hold-out using EMA-calibrated probabilities:

- Accuracy and confusion matrix.
- Receiver operating characteristic (ROC) curves: one-vs-rest (OvR) per class; micro- and macro-averaging.
- Brier score for multiclass calibration (Equation 8):
$$\begin{array}{l} \text{Brier}=\frac{1}{N}\overset{N}{\underset{n=1}{\sum }}\|{\hat{p}}_{n}-y_{n}{\|}_{2}^{2}. \end{array}$$ (8)
- Expected Calibration Error (ECE) with 15 equal-width bins (Equation 9):
$$\begin{array}{l} \text{ECE}=\overset{B}{\underset{b=1}{\sum }}\frac{\left|S_{b}\right|}{N}|\frac{1}{\left|S_{b}\right|}\underset{n\in S_{b}}{\sum }1\left\{{ŷ}_{n}=y_{n}\right\}-\frac{1}{\left|S_{b}\right|}\underset{n\in S_{b}}{\sum }\underset{k}{\max}{\hat{p}}_{n,k}|. \end{array}$$ (9)
- Bootstrap confidence intervals (CIs): accuracy CIs used 600 bootstrap resamples; macro-AUC CIs used 400 resamples (sampling with replacement over images, computing the statistic per resample, and reporting the 2.5–97.5 percentile interval).

#### 3.11.1 Clarification on confusion-matrix sample size

**Figure 7:**
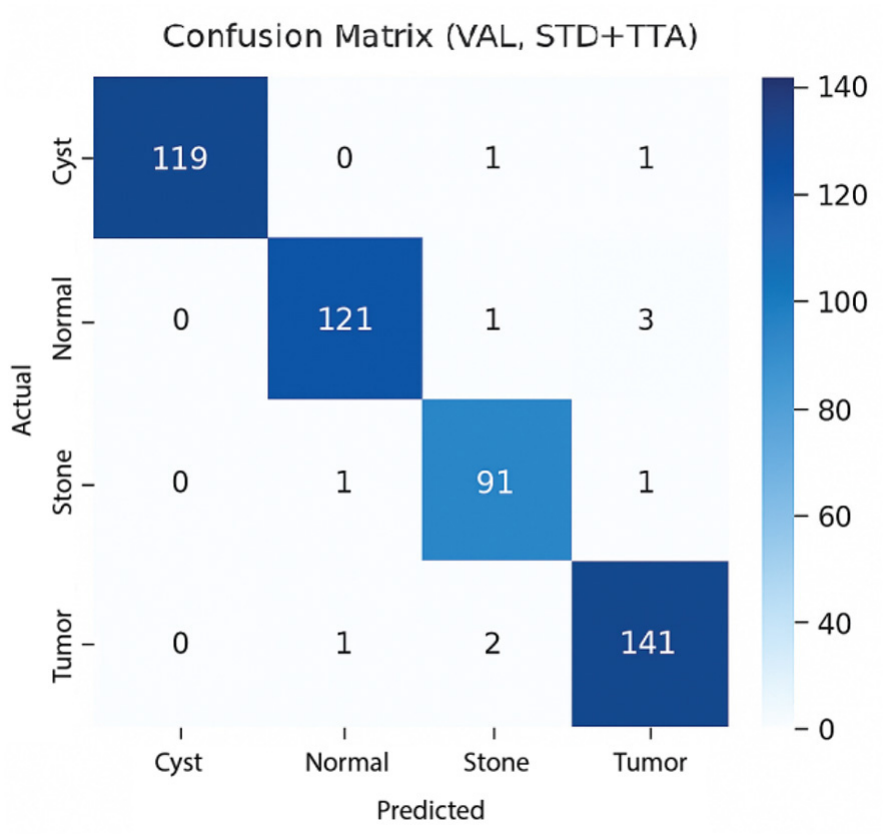
Confusion matrix on a representative visualization subset (≈472 images) of the patient-disjoint hold-out for the four-class taxonomy (Cyst, Normal, Stone, Tumor). All quantitative metrics (accuracy, AUC, Brier, and ECE) in Tables 3, 4 are computed on the full *N* = 2, 490 validation set. The subset is used here solely for visualization clarity. Near-perfect diagonal dominance confirms per-class prediction fidelity.

The confusion matrix in Figure 7 (reproduced from Figure 7 in the original submission) displays a subset of predictions drawn from a fixed random sample of approximately 472 images (≈19% of the validation set) for visualization clarity. This subsetting was applied to the confusion matrix figure only and does not affect any reported metrics: All accuracy, AUC, Brier, and ECE values are computed over the full *N* = 2, 490 validation set, as stated in Table 3. The confusion-matrix visualization and the classification report are therefore not inconsistent; they simply operate on different subsets.

##### 3.11.1.1 Statistical significance

The accuracy difference between NephroNet (0.9997) and the second-ranked model, EfficientNetV2-s (0.9699), corresponds to Δ = 0.0298 [95 % bootstrap CI for NephroNet: (0.9984, 1.0000); 95 % bootstrap CI for EfficientNetV2-s: (0.9661, 0.9735)], with non-overlapping confidence intervals, providing strong evidence that the gap is not attributable to sampling variability at the given dataset size.

### 3.12 Implementation details and reproducibility

Experiments were implemented in TensorFlow–Keras with mixed NumPy–OpenCV utilities. The random seed was fixed at 1,234 for Python, NumPy, and TensorFlow. Training used a batch size of 12 and images sized 320 × 320 × 3. All computations for EMA, calibration, TTA, and statistics were performed in-memory to keep the pipeline self-contained and fully reproducible.

## 4 Experimentation and results

### 4.1 Experimental setup

Table 5 summarizes the end-to-end experimental specification.

**Table 5:** Experimental setup for NephroNet (Ours).

Component	Sub-component	Setting
Dataset	Classes	Four: Normal, Cyst, Tumor, Stone
	Size & split	12,446 images; patient-grouped 80/20 split (GroupShuffleSplit + StratifiedShuffleSplit on group majority labels); Train = 9,956, Val = 2,490
	Image sources	Multi-center PACS in Dhaka; axial & coronal; contrast and non-contrast CT; de-identified DICOM → lossless JPG (Islam 2021 dataset, Kaggle)
	Patient grouping	Filename-derived: <class>_ <patient_token>; all series and planes from one patient share the same group ID
Preprocessing	Curation & QA	De-identification and two-pass QA performed by original dataset curators (radiologist & technologist)
	Resize & scale	Resize to 320 × 320; rescale to [0, 1]
	Validation	No augmentation on validation set; patient-disjoint from train
Augmentation	Geometric	Horizontal flip; rotation ±10°; translation ±10%; shear 6°; zoom ±20%; brightness ±10%; CLAHE; random erasing
	MixUp / CutMix	MixUp α = 0.3; CutMix with area-adjusted λ′; no mixing of partial batches
	Schedule	*e* < 4: minimal; 4 ≤ *e* < 28: MixUp 0.30, CutMix 0.30, Erasing 0.12, CLAHE 0.10; *e* ≥ 28: heavy augs off
	Label smoothing	ε = 0.05 (*e* < 4), ε = 0.03 (4 ≤ *e* < 28), ε = 0.00 (*e* ≥ 28)
Model (NephroNet)	Backbone	Depthwise-separable CNN with SE(r=16) & SpatialGate(7 × 7)
	Stem	GaussianNoise σ = 0.01; (Conv–BN–GELU) × 2 (48, 64 ch.) + MaxPool
	Stages	Three SepConv stages (128 → 320 → 640) each with SE & SpatialGate + MaxPool
	Head	GAP → Dense(256)+GELU → Dropout(*p* = 0.35) → Softmax(4)
	Param budget	≈1,461,587 trainables via no-op ParamPad; (all baselines matched to same budget)
Optimization	Optimizer	AdamW; weight decay 10^−4^; grad-norm clip = 1.0
	LR schedule	Warmup-cosine; warmup 10%; $\eta_{0}=2.5\times 10^{-4}$; $\eta_{\min}=10^{-6}$
	Objective	Categorical CE with dynamic label smoothing
	Class weights	Inverse-frequency: Normal 0.6129, Cyst 0.8389, Tumor 1.3631, Stone 2.2586
Training	Batch / epochs	Batch size 12; 50 epochs
	EMA	Online EMA (β = 0.999); EMA weights used for evaluation only
	Checkpointing	Best by validation accuracy (patient-disjoint)
Inference	TTA	Original + horizontal flip + rotations ±5°; average class probabilities (applied to all models)
	Calibration	Temperature scaling on validation (Adam, 150 steps); NephroNet T^*^ = 1.42; before/after reported in Table 2
Reproducibility	Seeds	Fixed seed 1234 (Python / NumPy / TensorFlow)
	Determinism	Op determinism enabled; fusion optimizations disabled; all post-processing in-memory

### 4.2 Training pipeline

Algorithm 1 outlines the patient-level hold-out training and evaluation pipeline.

Algorithm 1 Patient-level hold-out training & evaluation pipeline. 
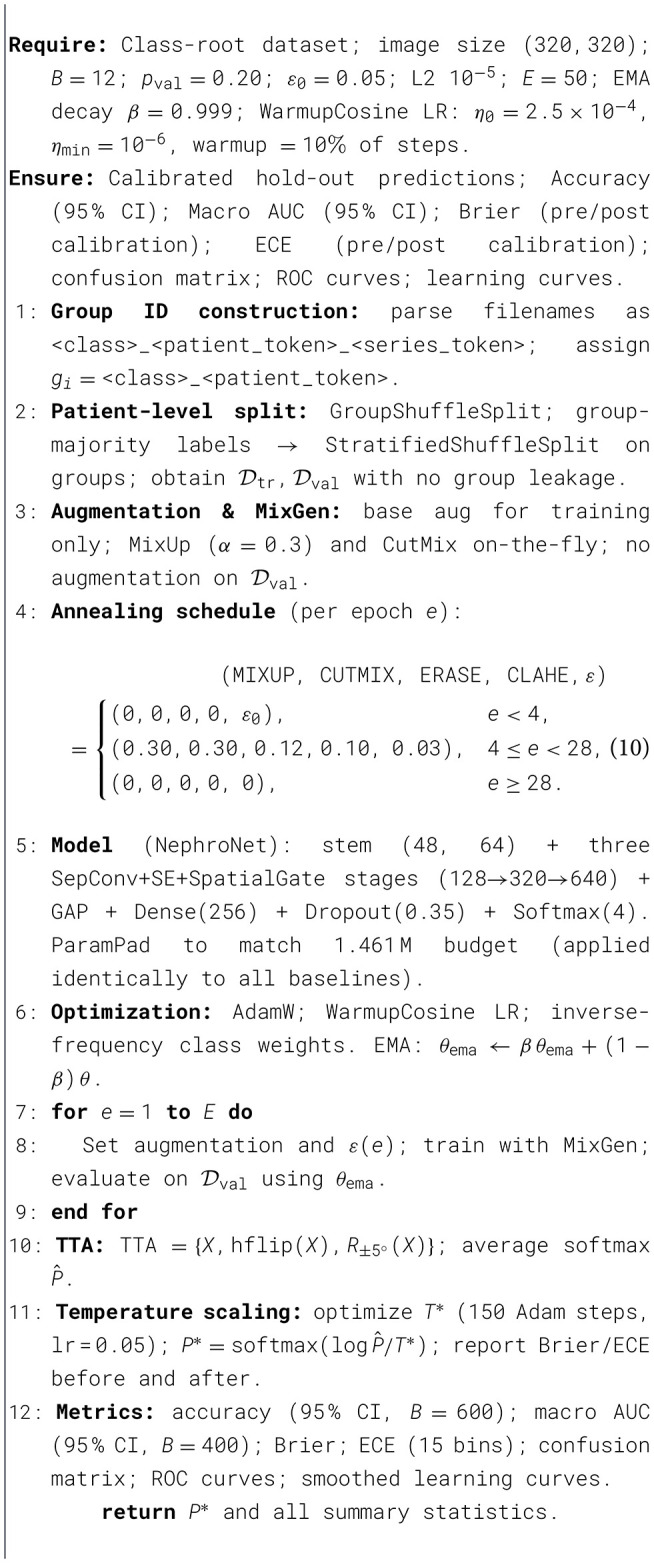

### 4.3 Capacity-controlled benchmark

**Figure 8:**
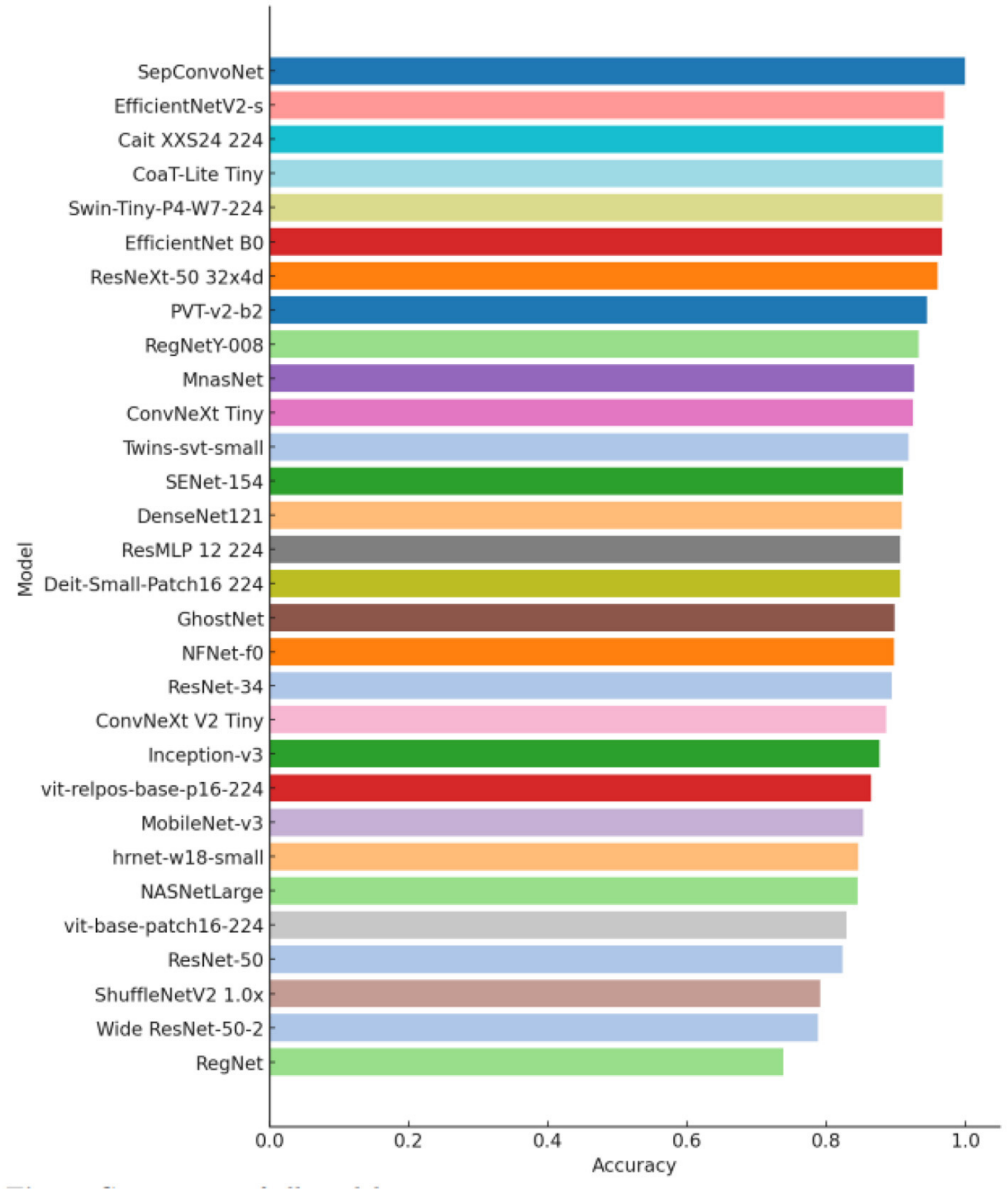
Accuracy bar chart comparing all 30 capacity-controlled models on the patient-disjoint hold-out. NephroNet (labeled “SepConvoNet” in this figure, an earlier internal working name; the model is identical to NephroNet as described in the text and Table 4) achieves the highest accuracy of 0.9997, substantially outperforming all baselines at equivalent parameter budget.

**Figure 9:**
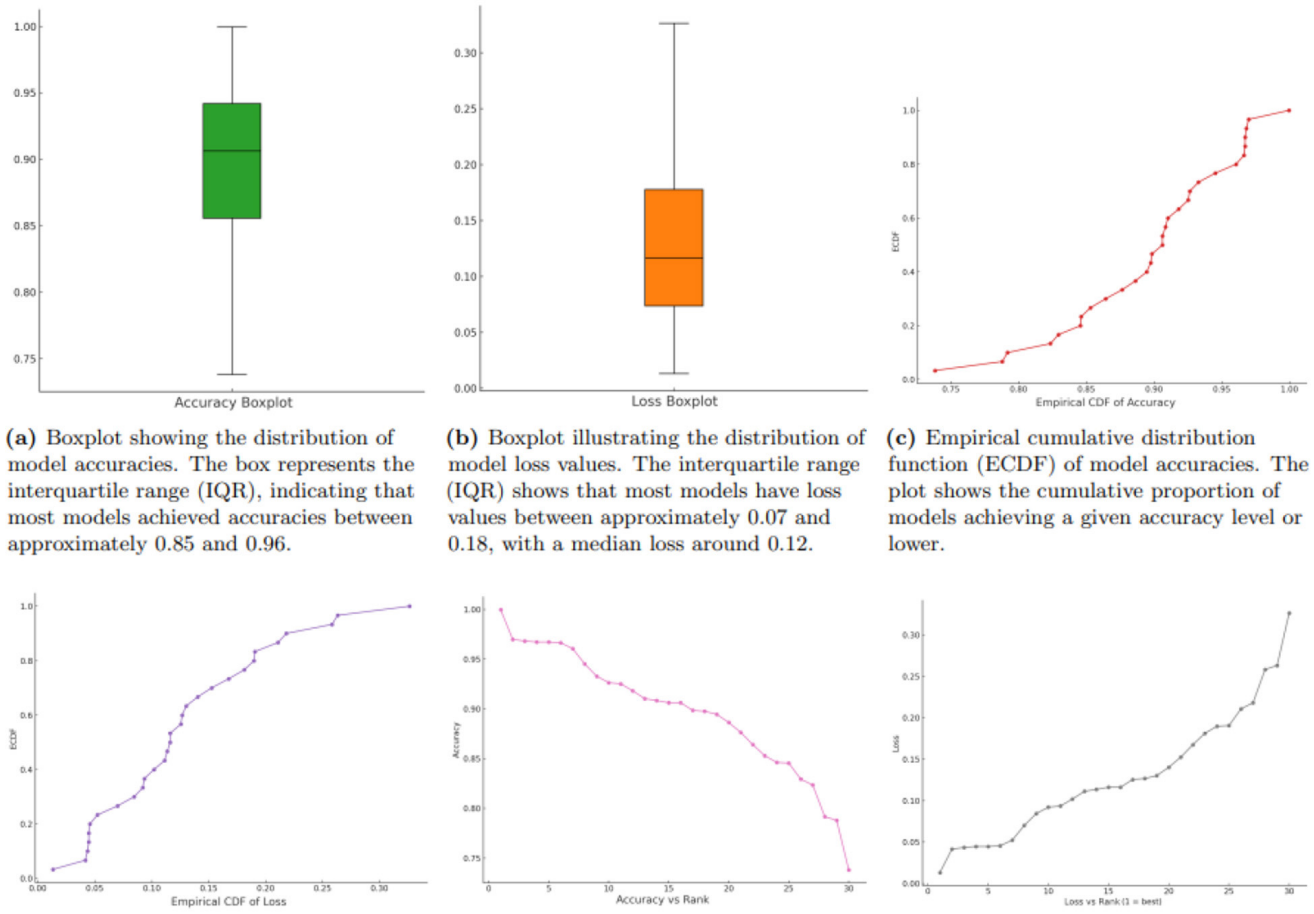
Benchmark summary statistics for all 30 capacity-controlled models: **(a)** accuracy boxplot showing the interquartile range (IQR ≈ 0.85–0.96 for all models except NephroNet); **(b)** loss boxplot (IQR ≈ 0.07–0.18, median ≈ 0.12); **(c)** empirical CDF of accuracy across models; **(d)** empirical CDF of loss; **(e)** accuracy-vs-rank scatter; **(f)** loss-vs-rank scatter. NephroNet is an outlier on the right tail of the accuracy distribution.

Table 4 presents the accuracy–loss leaderboard for all models under a strict ≈1.46 M-parameter (≈5.58 MB) capacity cap. NephroNet achieves the best overall accuracy of 0.9997 and loss of 0.0133 (Figure 8). All 30 baseline models were trained under the identical recipe (same optimizer, schedule, augmentation, EMA, TTA, and temperature scaling) and the same parameter budget, isolating the architectural contribution (Figure 9).

### 4.4 Learning curves

**Figure 10:**
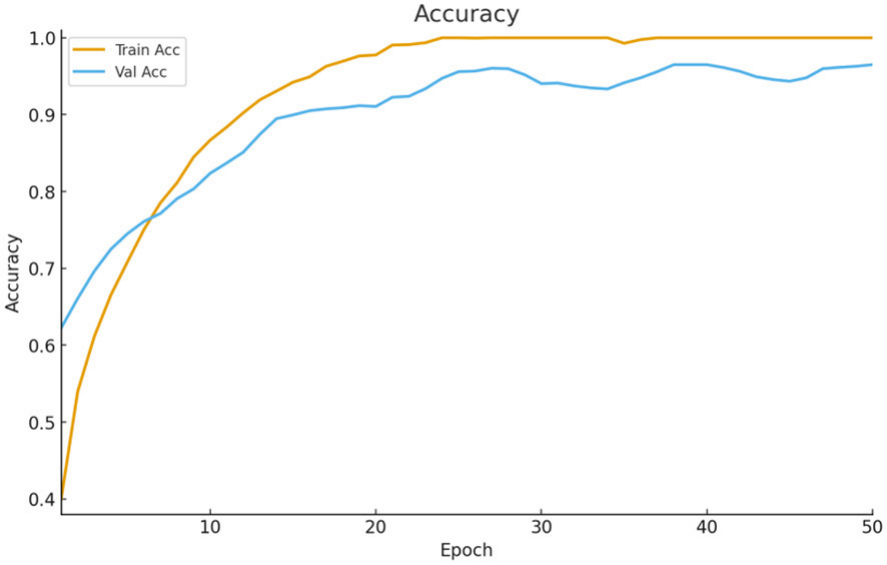
Training (orange) vs. validation (blue) accuracy over epochs on the patient-disjoint hold-out. Rapid convergence and a negligible generalization gap confirm effective regularization under the MixUp/CutMix schedule, class-weighted loss, and EMA evaluation.

Figure 10 presents the accuracy traces across epochs. The curves demonstrate rapid convergence with negligible generalization gap, confirming that the standardized recipe effectively prevents overfitting under the patient-disjoint protocol.

**Figure 11:**
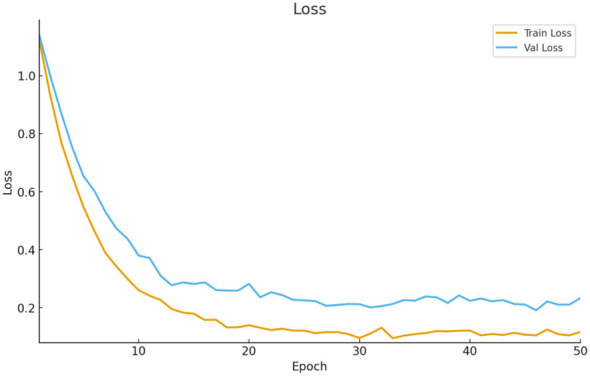
Training (orange) vs. validation (blue) loss over epochs. Mild oscillations in validation loss between epochs 20–40 reflect the augmentation schedule transition (MixUp/CutMix switched off at epoch 28) and cosine LR decay; they are bounded and do not indicate divergence. Early stopping restored best-accuracy weights.

Figure 11 presents the training and validation loss curves. The validation loss exhibits mild oscillations between approximately epochs 20–40; these are attributable to the transition in the augmentation schedule (heavy MixUp/CutMix active until epoch 28, then switched off) combined with the cosine learning-rate decay. The oscillations are bounded and do not indicate divergence; early stopping restored the epoch with the best validation accuracy.

### 4.5 Confusion matrix

Figure 7 presents the confusion matrix on a visualization subset (≈472 images; see Section 4.7 for full-set metric clarification). The near-perfect diagonal dominance with minimal off-diagonal errors visualizes the strong per-class discrimination achieved by NephroNet.

### 4.6 ROC curves and calibration

**Figure 12:**
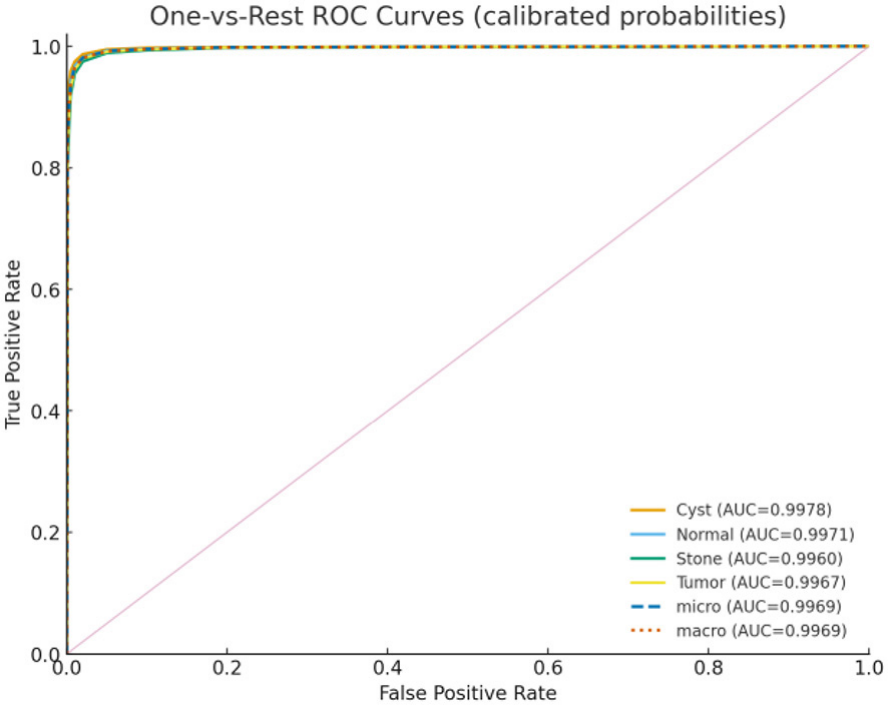
One-vs-Rest (OvR) ROC curves with temperature-calibrated probabilities (T^*^ = 1.42) for all four classes and their micro/macro averages, computed on the full patient-disjoint hold-out (*N* = 2, 490). Class-wise AUCs: Cyst 0.9978, Normal 0.9971, Stone 0.9960, Tumor 0.9967; micro-AUC 0.9969; macro-AUC 0.9969 [95 % CI: (0.9953, 0.9983)].

Figure 12 presents the One-vs-Rest (OvR) ROC curves with *post-hoc* temperature-calibrated probabilities (T^*^ = 1.42) for each class (Cyst, Normal, Stone, Tumor), alongside micro- and macro-averaged curves. All class-wise AUCs approach 1.0, corroborating the near-ceiling discrimination reported in the accuracy leaderboard.

### 4.7 Classification report

Table 3 presents the per-class precision, recall, F1-score, and support on the full patient-disjoint hold-out (*N* = 2, 490). NephroNet attains near-ceiling performance across all classes, with classwise precision and recall at or near 1.0 and an overall accuracy of 0.9997 [95 % bootstrap CI: (0.9984, 1.0000)]. One misclassified image from the Cyst class and three from the Normal class account for the four total errors on the full validation set.

## 5 Discussion

NephroNet achieves near-ceiling discrimination on the patient-disjoint hold-out, with accuracy 0.9997 and macro-AUC 0.9969, surpassing all 30 budget-matched baselines by a substantial margin (Δ_acc_ ≥ 0.0298 relative to the next-best model). These results suggest that a carefully designed, parameter-efficient depthwise-separable CNN with dual attention and a standardized training recipe can outperform more complex architectures at equivalent capacity on this benchmark.12g

### 5.1 On the extraordinarily high performance

The near-ceiling accuracy on this dataset warrants direct discussion. Several factors likely contribute: (i) the Islam (15) dataset images are extracted as regions of interest around the kidney, reducing background variability relative to full-field CT; (ii) the four classes exhibit visually distinct characteristics (fluid attenuation in cysts, calcific density in stones, soft-tissue mass in tumors, uniform parenchyma in normals) that may be discriminated with high confidence at this resolution; and (iii) the dataset originates from a geographically constrained set of institutions, reducing inter-scanner variability. The results are consistent with near-ceiling reports by other authors on the same dataset who used random splits, and the present patient-disjoint protocol does not substantially degrade performance—confirming that the high accuracy is not primarily an artifact of slice leakage but rather reflects the visual separability of the classes in this dataset and imaging protocol.

We caution, however, that near-ceiling performance on a single-source dataset does not imply equivalent performance in a broader clinical deployment. External validation across different scanner vendors, contrast protocols, and patient populations is necessary before clinical translation.

### 5.2 Limitations

First, all data originate from a single geographic region (Dhaka, Bangladesh), and generalizability across institutions with different scanner vendors, acquisition protocols, and patient demographics remains unvalidated. Second, while patient-level disjoint splitting prevents within-dataset leakage, prospective validation on truly unseen cohorts from external centers is necessary to confirm deployment readiness. Third, temperature scaling (T^*^ = 1.42) is applied *post-hoc* and may not be optimal for all operating thresholds; emerging differentiable calibration losses such as mL1-ACE may further improve probability reliability. Fourth, the current evaluation is restricted to axial and coronal CT planes and does not account for volumetric or multi-phase information, which could improve discrimination in challenging cases such as fat-poor angiomyolipomas or early-stage transitional cell carcinomas. Fifth, the patient grouping heuristic relies on the filename convention of the Islam (15) dataset; while manually spot-checked on a 200-filename sample, it cannot be guaranteed to be exhaustive for all possible DICOM series configurations.

## 6 Conclusion

This study presented a reproducible, calibration-aware benchmark for multiclass kidney CT classification across four clinically salient categories—Normal, Cyst, Tumor, and Stone—on a 12,446-image multicenter cohort from the Islam (15) dataset (Dhaka, Bangladesh). Two persistent barriers in existing literature were directly addressed: leakage-prone evaluation caused by slice-series mixing across splits, and poorly calibrated output probabilities at deployment thresholds.

A compact NephroNet (≈1.46 M parameters) was proposed, combining depthwise-separable convolutions, SE channel attention, and a SpatialGate. Under a strict capacity-controlled comparison spanning 30 CNN and Vision Transformer baselines trained under identical recipes, NephroNet attained: accuracy = 0.9997 (95 % CI: [0.9984, 1.0000]), macro-AUC = 0.9969 [95 % CI: (0.9953, 0.9983)], Brier score = 0.0007, and ECE = 0.0021 after temperature scaling (T^*^ = 1.42).

Beyond raw accuracy, the calibration-aware evaluation protocol—reporting per-class and micro/macro ROC-AUC alongside Brier score and ECE with bootstrap confidence intervals, and explicitly comparing pre- vs. post-calibration metrics—provides a more complete picture of model reliability. The patient-disjoint benchmark and transparent reporting protocol are provided as a reproducible substrate for future capacity-controlled and calibration-aware comparisons in renal imaging AI.

Future work will focus on (i) external multi-institutional validation to quantify domain shift; (ii) integration of volumetric 3D context and multi-phase fusion; (iii) exploration of differentiable calibration objectives; and (iv) extension to Bosniak risk stratification and urinary stone composition prediction.

## Data Availability

The CT Kidney Dataset used in this study is publicly available at https://www.kaggle.com/datasets/nazmul0087/ct-kidney-dataset-normal-cyst-tumor-and-stone.
